# Evaluation of the Manual Dexterity of Second-Year Dental Students after One Semester of Preclinical Training

**DOI:** 10.1055/s-0045-1811601

**Published:** 2025-09-11

**Authors:** Manar Qashou, Hana Mulhem, Deema Aldweik, Noor Najim, Asmaa T. Uthman, Mawada Abdelmagied, Tareq Aljafarawi, Musab Saeed, Natheer H. Al-Rawi

**Affiliations:** 1 College of Dental Medicine, University of Sharjah, Sharjah, United Arab Emirates; 2 Department of Diagnostic & Surgical Dental Sciences, College of Dentistry, Gulf Medical University, Ajman, United Arab Emirates; 3 Department of Basic Sciences, College of Dentistry, Ajman University, Ajman, United Arab Emirates; 4 Department of Clinical Sciences, College of Dentistry, Ajman University, Ajman, United Arab Emirates; 5 Centre of Medical and Bio-allied Health Sciences Research, Ajman University, Ajman, United Arab Emirates; 6 Research Institute of Medical and Health Science Research, University of Sharjah, Sharjah, United Arab Emirates

**Keywords:** manual dexterity, Pegboard Test, O'Connor test, dental training

## Abstract

**Objectives:**

Utilizing the Purdue Pegboard exam and the O'Connor exam, we assessed the impact of a semester-long preclinical training on the manual dexterity of dental students. Moreover, we examined the impact of gender, additional dental education, and practical exercises on manual dexterity.

**Materials and Methods:**

The study comprised a cohort of 45 preclinical students who were in their first year of dental school. The sample process was simple and convenient. Assessments were conducted at two distinct time points: T0, before the preclinical training laboratory, and T1, 7 months after the preclinical training laboratory. The Purdue Pegboard Test and the O'Connor Tweezer Dexterity Test were administered under identical conditions in both rounds of the trial. A validated survey was utilized to collect data on the gender, hands on activities, extra dental training, artistic skills, psychomotor skills, outdoor activities, and previous exposure to dental skills of each participant.

**Results:**

The dental students in the study demonstrated a significant improvement in their manual dexterity skills from the dental simulation preclinical laboratory training (T0) to 7 months of laboratory training (T1). This improvement was measured using the Purdue Pegboard Test and the O'Connor Tweezer Dexterity Test, with statistical significance at *p* < 0.05. Females have a significantly higher score in the Purdue indirect visual test of the left hand than males. Students who dedicated extra time to dental training showed significant improvements compared with those who did not allocate extra time for training.

**Conclusion:**

This study emphasizes the importance of manual dexterity in dental education and its correlation with preclinical training, hands-on practice, and gender. The results indicate significant improvements in manual dexterity following one semester of preclinical training, with discernible differences between genders. The findings highlight the significance of practice and further training in improving manual dexterity skills in dental students. This suggests that there may be implications for the development of curriculum and admissions procedures in dental education.

## Introduction

Manual dexterity refers to the ability to manipulate items and execute exact movements with coordination using one's hands. Possessing this competence is crucial for a dentist to effectively perform dental procedures with exactness and precision, resulting in conservative and aesthetically pleasing outcomes for patients. 1 Although the manual dexterity test is crucial for predicting the capacity of dental students in preclinical work, not all dental schools include this test in their admission process and are unaware of its value. This raises the question of whether manual dexterity testing should be considered equally important as grade point average (GPA) in the screening process, and whether it should be a universally adopted practice in all dental schools globally. 2

Novack and Turgeon conducted a study to determine whether the results of the dental aptitude test can predict preclinical grades in dental school. They discovered that students who scored 10 or higher on the manual dexterity test performed better in both preclinical and clinical courses. Therefore, they recommend using these scores as criteria in the admissions process. Furthermore, it was determined that raising the cut-off score of the exam could effectively filter out students who have difficulties in developing psychomotor skills. 3 The advantage of assessing and distinguishing the manual dexterity abilities of each student is to tailor their clinical experience to their individual abilities. In addition, students may experience dexterity concerns such as carpal tunnel syndrome, manual stiffness, pain, trembling, and impaired hand–eye coordination, all of which might impact the effectiveness of dental treatment. 1

In general, dentists require remarkable precision skills on a very small scale to carry out dental procedures. Therefore, it is crucial to include manual dexterity assessments in all dental schools. 4

Tests such as the Purdue Pegboard, O'Connor Finger Dexterity tests, and Functional Dexterity tests are frequently employed to assess the skills of dentistry students. 4 In 2022, Saeed et al utilized the Purdue Pegboard test and the O'Connor Tweezer test to assess the manual skill of dental students in their preclinical year. It was shown that both direct and indirect manual abilities showed a considerable increase with time, and gender did not have any impact on the extent of improvement. Furthermore, the utilization of indirect vision led to a decrease in student performance compared with the use of direct vision. Their findings suggest that additional training over a period of time can potentially improve manual dexterity. 5

Students' manual dexterity is influenced by their engagement in many activities, including painting, knitting, and playing musical instruments. Johnson et al suggest that if participants do not engage in these activities, it may indicate a slower development of their dental fine motor skills. To prevent these students from lagging behind their peers during simulation clinics, it is crucial to offer them guidance and hands-on tutorial instruction. 6 Multiple studies have established a connection between the lack of engagement in these activities before attending dental school and worse scores on psychomotor tests. This highlights the significance of hobbies and tasks such as jewelry-making that can enhance manual dexterity. 7

The objective of this study is to investigate whether engaging in preclinical practice in restorative laboratories, which involves the utilization of dental hand instruments with both direct and indirect vision, has an impact on the manual dexterity of dental students. The assessment of manual dexterity will be conducted on year 1 dentistry students during the spring semester of 2023, utilizing the Purdue Pegboard Test and the O'Connor Test. The assessments will be repeated with the same students at the commencement of the spring semester in the year 2024. Students' manual dexterity is influenced by their engagement in various hobbies, like painting, crocheting, and playing musical instruments, as previously noted. Therefore, this study will also investigate the impact of these attributes on their manual dexterity performance.

## Materials and Methods

### Participants

Before collecting data, the University Research Ethics Committee granted authorization for this project with the approval number (REC-23-01-23-01-S). After providing a thorough description of the study's objective and emphasizing the significance of keeping the data anonymous, we obtained the informed consent of the participants. The sample size ( *n* ) was obtained using G*Power version 3.1 software. The *t* -test statistical family parameter was used, assuming a medium effect size of 0.5, an *α* error probability of 0.05, and a study power of 0.9. The minimum sample size was 45. Hence, a total of 45 dental students, who were in their first year at the university, voluntarily agreed to take part in this study. They were then monitored until the second semester of the next academic year. Prior to commencing the trial, all participants provided their signature on an informed consent document.

### Purdue Pegboard Test

The test comprises a board with two columns of holes. At the distal end of the board, there are four cups. These cups consist of a right cup and a left cup, each carrying 25 pins. Additionally, there is a cup with washers and a cup with collars. There are four tasks that need to be completed in this test. The initial three activities necessitate the insertion of pins into holes on the board within a time limit of 30 seconds, while the final task necessitates a time limit of 60 seconds. The score of every task is determined by the number of pins that are inserted. The roles display disparities in the following manner: The third task was completed by first using the dominant hand and then the nondominant hand, involving both hands in the process. This required holding one pin in each hand and inserting them simultaneously into two corresponding holes.

The fourth task is the Purdue direct assembly. During the assembly process, the participant consistently utilizes both hands to put together four components. The process begins with inserting a pin, followed by threading a washer and a collar, and concludes with another washer. Following the completion of the four tasks using direct vision, the subjects proceeded to execute the same four tasks utilizing indirect vision.

**Fig. 1:**
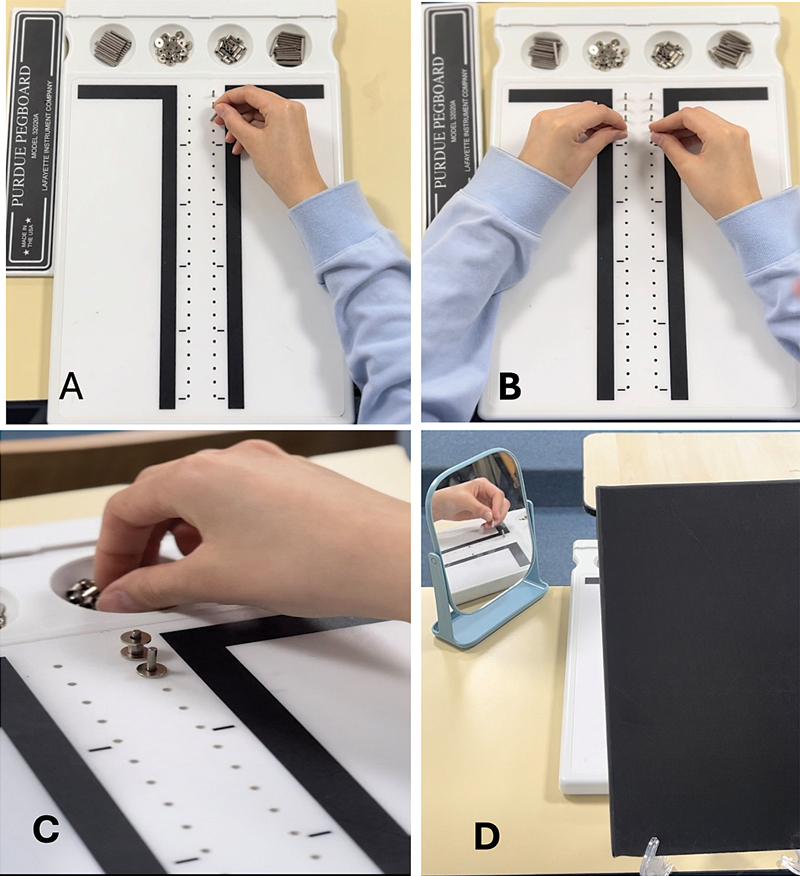
Purdue Pegboard Test has four tasks. First and second tasks are done by using the dominant and nondominant hands ( **A** ) and then by using both hands ( **B** ) and the fourth task (assembly task) ( **C** ). These tasks should be conducted twice, first by using direct vision ( **A–C** ) and then indirect vision using mirror and a blackboard shield to hide the board test ( **D** ).

The participant sequentially threads a washer and a collar, assembles four components (commencing with a pin), and concludes by threading another washer, all while maintaining continuous use of both hands. After completing the four activities using direct vision, the individuals proceeded to do the same four tasks using indirect vision ( Fig. 1 ).

### O'Connor Tweezer Test of Dexterity

**Fig. 2:**
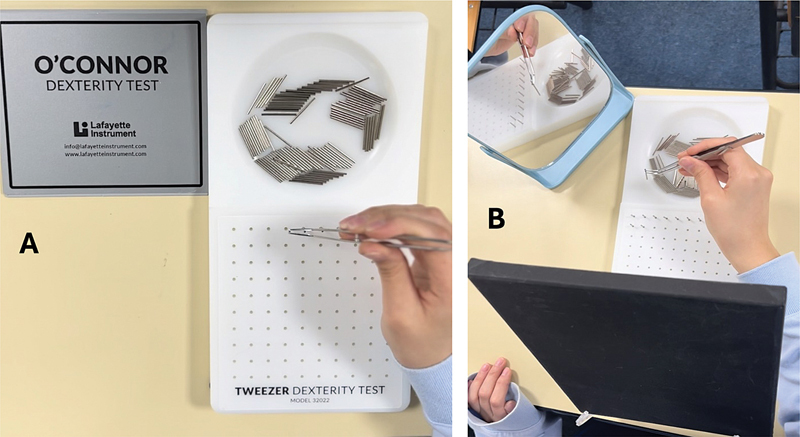
( **A, B** ) Direct and indirect visions for O'Connor Dexterity Test.

This test consists of a board with 100 holes and a cup with 100 pins. The participant meticulously positions each of the 100 pins using tweezers and their dominant hand. During this examination, O'Connor Tweezers were used for both direct and indirect vision. The duration required to insert each pin into a hole was established as 5 minutes, and the score was computed accordingly ( Fig. 2 ).

To establish indirect vision for both the Purdue and O'Connor tests, a blackboard shield is used to cover the test board. This prevents participants from directly seeing the board and allows them to complete activities using a mirror. The assessment was conducted in a calm, quiet, and meticulously maintained environment to eliminate any potential distractions. Prior to commencing each level, all participants were provided with consistent and clear direction by a single instructor via written notes. Students were given the freedom to take brief breaks, enabling them to extend their legs by moving around the classroom in between tasks. Evaluations were performed at two specific points in time: T0, prior to the preclinical training laboratory, and T1, following the preclinical training laboratory, which occurred 7 months after T0.

### Questionnaire

We have utilized a validated questionnaire that was previously employed in another investigation. 6

**Fig. 3:**
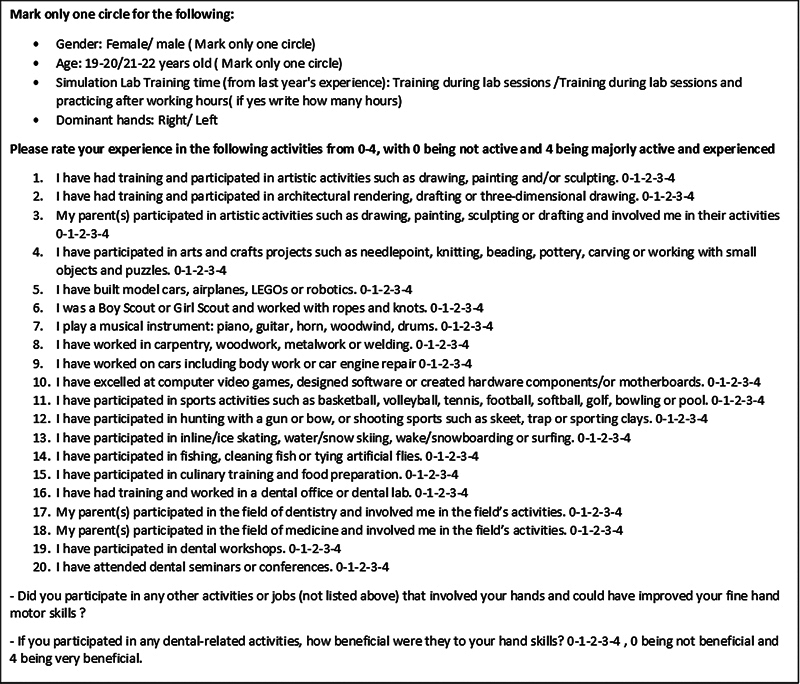
A questionnaire composed of 26 questions about gender, dominant hand, extra dental training, artistic skills, psychomotor skills, outdoor activities, and prior experience on participants' dental skills.

The questionnaire consists of 25 questions pertaining to gender, hands-on activities, extra dental training, artistic skill, psychomotor skills, outdoor activities, and prior expertise in the participant's dental skills. The background of each participant in terms of gender, age, and training time in the simulation preclinical training laboratory is obtained through four questions. Fill-in section at the end of the question was provided to write how many hours they spent, dominant hand (right or left). The remaining 20 questions pertain to the extent of training and lifelong engagement with diverse physical activities prior to dental school. These activities encompass visualization as well as the development of both gross and fine motor skills, such as artistic drawing, painting, arts and crafts, participation in boy and girl scouts, playing musical instruments, carpentry, car repair, team and individual sports, hunting, fishing, cooking training, and working in dental or medical offices or laboratories. For each activity, participants need to provide a rating from 0 to 4, with 0 indicating no involvement and 4 indicating the highest level of involvement, based on their lifelong participation. At the conclusion of the survey, there was a place where students could provide additional activities that were not included in the provided list, as depicted in Fig. 3 .

### Statistical Analysis

Quantitative variables are represented by means and standard deviation (SD), while qualitative variables are presented as counts in percentages. To assess normality, the Shapiro–Wilk test was employed. Spearman's correlation was one of the nonparametric tests utilized to examine the relationship between the survey domain and the skills measurements of the participants. For normal and nonnormal variables, we compared pre- and post-participant skill measurements using the paired *t* -test and Wilcoxon's rank test, respectively. Utilizing the Spearman correlation coefficient (rho), both the strength and the direction of the relationship were determined. The investigators employed multivariate linear regression analysis to predict the impact of prior experience, artistic skill, psychomotor skills, hands-on activities, and artistic skill on the dental skills of the participants. Differences between groups, correlations, and effects of predictors were considered statistically significant at *p* < 0.05. Statistical analysis was performed using the statistical package for the social sciences (SPSS) computer software (version 27), IBM software, United States.

## Results

**1.** The study included a cohort of 45 dental students, with females comprising a greater proportion than males (57.8 vs. 42.2%). Approximately 71.1% of the participants were right-handed, whereas the remaining 28.9% were left-handed. Just 35.6% of the participants dedicated extra hours to improve their manual dexterity ( Table 1 ).

**Table 1:** Descriptive statistics of gender, hands-on activities, and extra dental training

Items	No. (count)	%
**Gender**		
Male Female	19 26	42.2 57.8
Dominant hand (hands-on activities)		
Right Left	32 13	71.1 28.9
Simulating laboratory training (extra dental training)		
During laboratory Laboratory + after working hours	29 16	64.4 35.6

**2. Test of normality** :

Applying the Shapiro–Walik test to the 20 variables under examination, it was found that 12 of them exhibited nonnormal distribution with a significance level of *p* < 0.05. Conversely, eight variables showed normal distribution, as indicated in Table 2 . Out of the 16 factors tested in the Purdue Pegboard Test, 9 showed statistical significance, with *p* -values ranging from 0.002 to 0.047. In the O'Connor Tweezer Test, three out of four variables showed statistical significance, with *p* -values ranging from <0.001 to 0.013.

**Table 2:** Test of normality using the Shapiro–Wilk test

Test	Statistics	Significance
Direct visual test of the right hand before training	0.965	0.191
Direct visual test of the right hand after training	0.968	0.247
Direct visual test of the left hand before training	0.909	0.002 a
Direct visual test of the left hand after training	0.946	0.036
Direct visual test of both hands before training	0.949	0.047 b
Direct visual test of both hands after training	0.907	0.002 a
Direct visual test of assembly before training	0.944	0.031 b
Direct visual test of assembly after training	0.959	0.108
Indirect visual test of the right hand before training	0.972	0.343
Indirect visual test of the right hand after training	0.960	0.120
Indirect visual test of the left hand before training	0.925	0.006 a
Indirect visual test of the left hand after training	0.965	0.183
Indirect visual test of both hands before training	0.947	0.04 b
Indirect visual test of both hands after training	0.942	0.026 b
Indirect visual test of assembly before training	0.930	0.009 a
Indirect visual test of assembly after training	0.932	0.011 b
Direct visual test O'Connor before training	0.980	0.602
Direct visual test O'Connor after training	0.847	< 0.001 a
Indirect visual test O'Connor before training	0.869	< 0.001 a
Indirect visual test O'Connor after training	0.934	0.013 b

a *p* < 0.01.

b *p* < 0.05.

**3. Pre- (T0) and post-training (T1) results:**

Table 3 presents the outcomes of the laboratory training pertaining to the manual dexterity skills of the students. Following dental preclinical laboratory training (T0), participants' skills in all parameters, including direct and indirect visual testing of the O'Connor Tweezers Test and the Purdue direct and indirect visual tests of the right, left, both hands, and assembly, increased significantly. Students' performance on the Purdue right-hand direct visual test (DVT) improved significantly ( *p* = 0.014), from ∼14 pins placed at T0 to 16 pins placed at T1. This finding aligns with the results of the Purdue right-hand indirect visual test (IVT), as the number of pins affixed at T1 increased from 13 to 15 in a statistically significant way ( *p* < 0.001).

**Table 3:** Comparison between the participants' skills at the beginning (T0) and after 7 months of the preclinical training course (T1)

Parameter	Pre-training (mean ± SD)	Post training (mean ± SD)	Test statistics value	*p* -Value
Direct visual test of the right-hand	14.69 ± 1.86	15.67 ± 2.13	2.568	0.014 a
Direct visual test of the left-hand	13.00 ± 1.55	14.20 ± 1.94	3.550	< 0.001 a
Direct visual test of both hands	10.93 ± 1.57	11.69 ± 1.77	2.431	0.015 a
Direct visual test assembly	7.40 ± 1.50	8.56 ± 1.78	2.882	0.004 a
Indirect visual test of the right-hand	5.60 ± 2.41	9.78 ± 1.86	9.335	< 0.001 a
Indirect visual test of the left-hand	4.82 ± 2.27	9.29 ± 1.87	5.664	< 0.001 a
Indirect visual test of both hands	4.02 ± 1.88	6.11 ± 1.42	4.604	< 0.001 a
Indirect visual test assembly	4.22 ± 1.36	5.13 ± 1.49	3.254	0.001 a
Direct visual test O'Connor	76.20 ± 12.74	87.64 ± 13.13	4.324	< 0.001 a
Indirect visual test O'Connor	15.64 ± 12.74	26.78 ± 10.41	4.971	< 0.001 a

Notes: Statistical analysis was done using paired *t* -test or Wilcoxon's test according to normal distribution analysis by Shapiro–Wilk test. The test statistics is *t* -value for the paired *t* -test or *z* -value for the Wilcoxon test.

a Significantly different at *p* < 0.05 ( *n* = 45).

The mean number of pins placed during the O'Connor Tweezers Test with direct vision was ∼76 over the course of 5 minutes. At T1, this number increased significantly ( *p* < 0.001) to 88 out of 100. Additional analysis reveals that the O'Connor Indirect Test improved significantly ( *p* < 0.001) from 16 at T0 to 27 pins at T1.

**4. Gender differences:**

During the Purdue Pegboard Test, females exhibited higher mean differences than males when both hands and the right hand were used in direct and indirect visual tasks. Furthermore, females exhibited higher scores in the left-hand IVT test (5.15 ± 2.56) and indirect assembly test (1.15 ± 1.59) compared with males (3.53 ± 2.57 and 0.58 ± 1.64), respectively. In general, females had better average scores in all indirect visual tests compared with males. In contrast, males exhibited greater mean differences than females in the left-hand DVT test (1.58 ± 2.32 vs. 0.92 ± 1.76) and direct visual assembly test (1.32 ± 2.79 vs. 1.04 ± 2.25). However, the only test that demonstrated a statistically significant difference ( *p* < 0.05) in skill outcomes between males and girls is the (IVT) of the left hand, with a *p* -value = 0.04. These findings suggest that females outperform males in the Purdue IVT of the left hand, with a statistically significant difference in scores.

Concerning the O'Connor's test, the direct vision test revealed a slightly greater mean difference value in males (72.05 ± 16.10) compared with females (71.96 ± 16.15). On the other hand, females exhibited higher average difference values in the indirect vision test than males (12.57 ± 10.51 vs. 9.16 ± 12.69). Nevertheless, the O'Connor Tweezer Test reveals no significant difference in skill results between males and females ( *p* > 0.05) ( Table 4 ).

**Table 4:** Descriptive statistics of participants' skills (difference between pre- and post-measurements) in relation to gender

Parameter	Male (mean ± SD)	Female (mean ± SD)	*p* -Value
Direct visual test of the right-hand	0.80 ± 2.72	1.12 ± 2.47	0.69
Direct visual test of the left-hand	1.58 ± 2.32	0.92 ± 1.76	0.30
Direct visual test of both hands	0.47 ± 1.98	0.96 ± 1.87	0.40
Direct visual test assembly	1.32 ± 2.79	1.04 ± 2.25	0.72
Indirect visual test of the right-hand	4.42 ± 3.54	4.00 ± 2.59	0.66
Indirect visual test of the left-hand	3.53 ± 2.57	5.15 ± 2.56	0.04 a
Indirect visual test of both hands	1.84 ± 2.14	2.27 ± 2.53	0.54
Indirect visual test assembly	0.58 ± 1.64	1.15 ± 1.59	0.25
Direct visual test O'Connor	72.05 ± 16.10	71.96 ± 16.15	0.98
Indirect visual test O'Connor	9.16 ± 12.69	12.57 ± 10.51	0.34

a *p* < 0.05.

### Dominant Hand Differences

The mean differences in the DVT of the right hand were higher in right-handed participants (1.00 ± 2.55) compared with left-handed ones (0.92 ± 2.66). Interestingly, the left-handed participants (2.77 ± 1.92) exhibited more pronounced differences compared with the right-handed participants (0.56 ± 1.70) while utilizing DVT of their left hands. This pattern also holds true when both hands were examined, with left-handed subjects (1.23 ± 1.69) showing greater differences than right-handed participants (0.56 ± 1.98). Left-handed participants had significantly higher mean difference values in both direct and indirect assembly tests. However, the mean differences were higher in right-handed participants when they were assessed using the direct O'Connor's test (72.22 ± 14.56 vs. 71.46 ± 19.58) and the indirect O'Connor's test (11.94 ± 8.96 vs. 9.15 ± 16.40). The only test that exhibited a significant difference in skill results between individuals who are right-handed and those who are left-handed was the DVT of the left hand. Furthermore, the scores of left-handed individuals in the Purdue DVT of the left hand are considerably greater compared with those of right-handed participants ( *p* -value = 0.0004). The results of the right-hand DVT suggest that there was no statistically significant difference in scores between individuals who are right-handed and those who are left-handed ( *p* = 0.92; Table 5 ).

**Table 5:** Descriptive statistics of participants' skills (difference between pre- and post-measurements) in relation to dominant hands used

Parameter	Right (mean ± SD)	Left (mean ± SD)	*p* -Value
Direct visual test of the right-hand	1.00 ± 2.55	0.92 ± 2.66	0.92
Direct visual test of the left-hand	0.56 ± 1.70	2.77 ± 1.92	0.0004 a
Direct visual test of both hands	0.56 ± 1.98	1.23 ± 1.69	0.29
Direct visual test assembly	1.00 ± 2.32	1.54 ± 2.84	0.51
Indirect visual test of the right-hand	3.97 ± 2.91	4.69 ± 3.27	0.47
Indirect visual test of the left-hand	4.81 ± 2.90	3.62 ± 1.76	0.18
Indirect visual test of both hands	2.16 ± 2.33	1.92 ± 2.53	0.76
Indirect visual test assembly	0.88 ± 1.60	1.00 ± 1.73	0.82
Direct visual test O'Connor	72.22 ± 14.56	71.46 ± 19.58	0.89
Indirect visual test O'Connor	11.94 ± 8.96	9.15 ± 16.40	0.46

Note: ( *n* = 45), 32 right, and 13 left.

a *p* < 0.001.

**5. Extra training effect:**

Twenty-nine students participated in laboratory training and 16 students completed laboratory training with extra after-work training. There was a noticeable improvement in the results of the DVT of the left hand among participants with extra training (2.31 ± 2.21) compared with only laboratory training (0.59 ± 1.64). This pattern is also present in the DVT of both hands (1.13 ± 1.74 vs. 0.55 ± 1.99), DVT assembly (1.25 ± 2.35 vs. 1.10 ± 2.57), IVT of the left hand (5.19 ± 2.93 vs. 4.07 ± 2.46), IVT of both hands (2.13 ± 2.78 vs. 2.07 ± 2.15), IVT assembly (1.19 ± 2.01 vs. 0.76 ± 1.38), and IVT O'Connor (12.25 ± 16.28 vs. 10.52 ± 7.98). The only variables that did not show improvement in scores were the DVT of the right hand, the IVT of the right hand, and the DVT of the O'Connor Test ( Table 6 ). Nonetheless, the only test that showed a statistically significant difference in skill results after extra laboratory training was the DVT of the left hand ( *p* = 0.0048).

**Table 6:** Descriptive statistics of participants' skills (difference between pre- and post-measurements) in relation to extra dental training

Parameter	During laboratory (mean ± SD)	Laboratory + after working hours (mean ± SD)	*p* -Value
Direct visual test of the right-hand	1.10 ± 2.64	0.75 ± 2.46	0.66
Direct visual test of the left-hand	0.59 ± 1.64	2.31 ± 2.21	0.0048 a
Direct visual test of both hands	0.55 ± 1.99	1.13 ± 1.74	0.33
Direct visual test assembly	1.10 ± 2.57	1.25 ± 2.35	0.85
Indirect visual test of the right-hand	4.31 ± 2.90	3.94 ± 3.26	0.70
Indirect visual test of the left-hand	4.07 ± 2.46	5.19 ± 2.93	0.18
Indirect visual test of both hands	2.07 ± 2.15	2.13 ± 2.78	0.94
Indirect visual test assembly	0.76 ± 1.38	1.19 ± 2.01	0.40
Direct visual test O'Connor	72.69 ± 16.55	70.75 ± 15.23	0.70
Indirect visual test O'Connor	10.52 ± 7.98	12.25 ± 16.28	0.63

Note: ( *n* = 45), 29 during laboratory, and 16 laboratory + after working hours.

a *p* < 0.001, domains were mildly engaged with by the participants as they ranged between 0.80 and 1.59.

**6. Participant's skills experience:**

The questionnaire in Table 7 was used to assess four dimensions of participants' skills. The responses were classified as “not engaged” when the Likert score fell within the range of 0 to 0.79. They were classified as “mildly engaged” when the score was between 0.80 and 1.59, and as “moderately engaged” when it fell within the range of 1.60 to 2.39. Finally, responses were classified as “actively engaged” when the score ranged from 4.20 to 5.0.

**Table 7:** Means and standard deviation of skills domains of the survey

Domain	Mean ± SD
Artistic skills	1.23 ± 1.08
Psychomotor skills	0.84 ± 0.99
Outdoor activities	0.87 ± 0.98
Prior experience	1.11 ± 0.91

To be more precise, around 43.7% of the participants did not possess creative talents, whereas only 11.65% of the participants were highly involved in artistic skills. Approximately two-thirds of the participants lacked psychomotor skills, with an average score of 62.13 ± 14.94. Only 5% of the individuals demonstrated a high level of engagement in this particular skill set.

Over 50% of the participants did not engage in any outside activities such as fishing, sports, woodworking, or hunting. Only 5% of the participants were highly engaged in outdoor activities.

In terms of previous dental experience, 64.3% of the participants reported having no prior experience, while just 10% indicated a high level of engagement with dental procedures in the past.

**Fig. 4:**
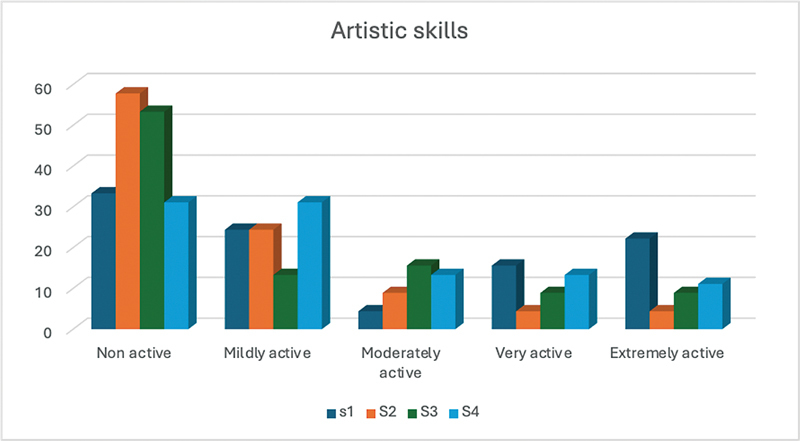
Frequency distribution of artistic skills.

**Fig. 5:**
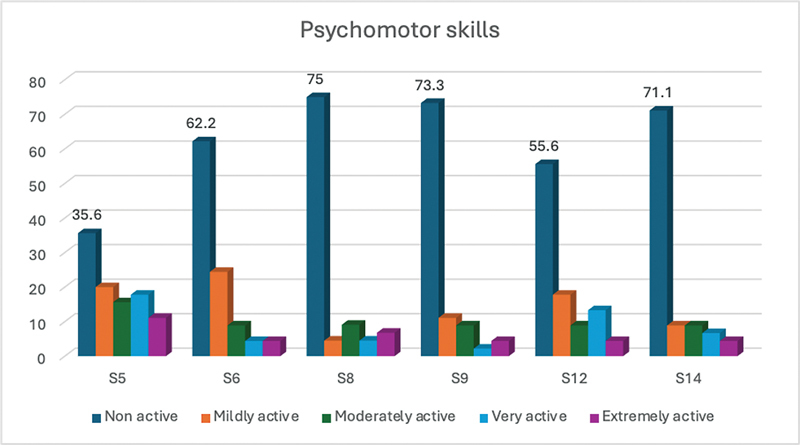
Frequency distribution of psychomotor skills.

**Fig. 6:**
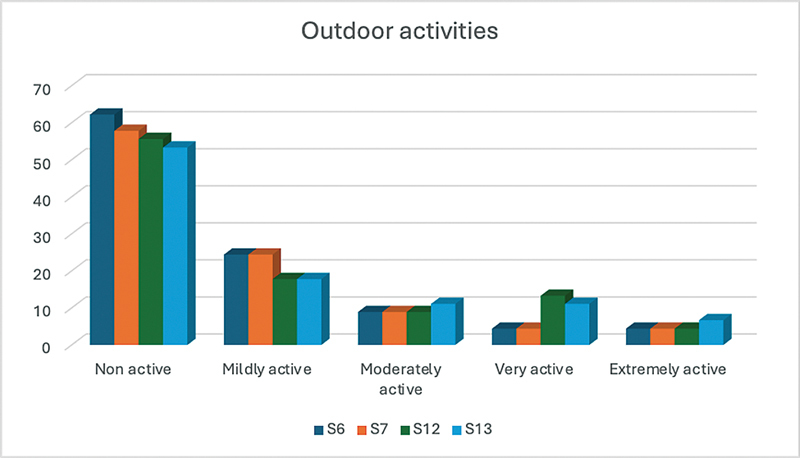
Frequency distribution of outdoor activities.

**Fig. 7:**
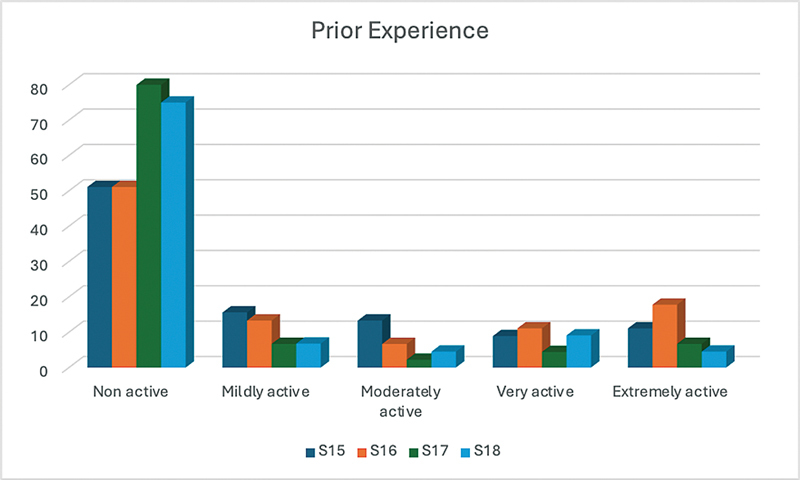
Frequency distribution of prior dental experience.

Figs. 4 to 7 demonstrate the frequency distribution of the aforementioned skills.

**7. Correlation between survey domains and participant's skills:**

The relationship between the survey domains and the skills of the participants, as measured by the difference between the pre- (T0) and post-measurements (T1), is displayed in Table 8 . There is a positive correlation between artistic skills and psychomotor skills ( *r* = 0.443; *p* = 0.002) as well as past experience ( *r* = 0.443; *p* = 0.002). Psychomotor skills exhibit a positive correlation with artistic skills ( *r* = 0.443; *p* = 0.002), outdoor activities ( *r* = 0.761; *p* = 0.000), and past experience ( *r* = 0.377; *p* = 0.011). Engaging in outdoor activities is positively correlated with the development of psychomotor skills ( *r* = 0.761; *p* < 0.001) and is also influenced by prior experience ( *r* = 0.463; *p* = 0.001). Previous experiences had a favorable correlation with artistic skills ( *r* = 0.443; *p* = 0.002), psychomotor skills ( *r* = 0.377; *p* = 0.011), and outdoor activities ( *r* = 0.463; *p* = 0.001). The psychomotor activities had a highly significant positive link with outdoor activities, as indicated by a strong correlation coefficient ( *r* = 0.761).

**Table 8:** Correlation between survey domains and participants' skills (difference between pre- and post-measurements)

		Artistic skills	Psychomotor skills	Outdoor activities	Prior experience
*Artistic skills*	Correlation coefficient (rho)				
	*p* -Value				
*Psychomotor skills*	Correlation coefficient (rho)	0.443			
	*p* -Value	0.002 a			
*Outdoor activities*	Correlation coefficient (rho)	0.334	0.761		
	*p* -Value	0.025 a	< 0.001 a		
*Prior experience*	Correlation coefficient (rho)	0.443	0.377	0.463	
	*p* -Value	0.002 a	0.011 a	0.001 a	
*Direct visual test of* the *right-hand*	Correlation coefficient (rho)	0.109	- 0.078	- 0.094	0.037
	*p* -Value	0.478	0.609	0.538	0.810
*Direct visual test of* the *left-hand*	Correlation coefficient (rho)	− 0.021	− 0.047	− 0.055	− 0.042
	*p* -Value	0.892	0.762	0.721	0.785
*Direct visual test of both hands*	Correlation coefficient (rho)	0.100	− 0.060	− 0.100	0.047
	*p* -Value	0.515	0.695	0.514	0.757
*Direct visual test assembly*	Correlation coefficient (rho)	0.140	− 0.103	− 0.080	0.029
	*p* -Value	0.358	0.499	0.599	0.851
*Indirect visual test of* the *right-hand*	Correlation coefficient (rho)	− 0.127	0.038	0.048	0.012
	*p* -Value	0.405	0.807	0.754	0.937
*Indirect visual test of* the *left-hand*	Correlation coefficient (rho)	0.174	− 0.104	− 0.177	0.108
	*p* -Value	0.254	0.496	0.245	0.481
*Indirect visual test of both hands*	Correlation coefficient (rho)	0.101	− 0.062	− 0.157	− 0.037
	*p* -Value	0.510	0.686	0.304	0.807
*Indirect visual test assembly*	Correlation coefficient (rho)	− 0.137	− 0.224	− 0.135	0.163
	*p* -Value	0.369	0.140	0.375	0.285
*Direct visual test of* O'Connor	Correlation coefficient (rho)	− 0.128	0.038	0.041	− 0.035
	*p* -Value	0.403	0.807	0.791	0.821
*Indirect visual test of* O'Connor	Correlation coefficient (rho)	− 0.131	− 0.107	0.037	− 0.038
	*p* -Value	0.392	0.483	0.809	0.802

Note: Spearman's correlation was used.

a Significant correlation at *p* < 0.05.

Nevertheless, the majority of correlation coefficients between visual test performance and examined domains such as artistic skills, psychomotor skills, outdoor activities, and prior experience are nearly nil, suggesting a weak or nonexistent association. Furthermore, there was no significant link between the skills categories and the participants' direct and indirect visual assessments ( *p* > 0.05). This implies that performance on visual tests is not significantly affected by criteria such as artistic skill, motor skills, outdoor activities, or previous experience, as indicated by the survey.

**8. The effect of predictors on direct visual test (T0 vs. T1):**

A multivariate linear regression analysis was performed to examine the impact of predictors on direct visual tests, namely, the difference between pre- and post-measurements. The predictors, such as gender, dominant hand, extra dental laboratory training, artistic skills, psychomotor skills, outdoor activities, and prior experience, did not have a significant impact on the results of the direct visual test on the right hand, both hands, assembly, and O'Connor test ( *p* > 0.05; Table 9 ).

**Table 9:** Multivariate linear regression analysis to study the effect of predictors on the direct visual test (difference between pre- and post-measurements)

	Predictors	Unstandardized coefficients	Standardized coefficients	*t* -Value	*p* -Value	*R* ^2^
Right hand	Male	− 0.001	0.000	0.001	1.000	0.048
	Right dominant hand	− 0.453	-0.081	0.428	0.671	
	Laboratory + after working hours	− 0.932	0.177	0.870	0.390	
	Artistic skills	0.481	0.203	0.905	0.371	
	Psychomotor skills	− 0.826	− 0.321	0.625	0.536	
	Outdoor activities	0.104	0.040	0.086	0.932	
	Prior experience	0.291	0.104	0.453	0.653	
Left hand	Male	0.414	0.102	0.633	0.530	0.360
	Right dominant hand	− 1.648	− 0.347	2.406	0.021 a	
	Laboratory + after working hours	1.435	0.344	2.066	0.046 a	
	Artistic skills	− 0.176	− 0.094	0.512	0.612	
	Psychomotor skills	0.253	0.125	0.296	0.769	
	Outdoor activities	− 0.633	− 0.307	0.804	0.427	
	Prior experience	0.115	0.052	0.275	0.785	
Both hands	Male	− 0.474	− 124	0.646	0.522	0.100
	Right dominant hand	− 0.957	− 0.230	1.246	0.221	
	Laboratory + after working hours	0.057	0.014	0.073	0.942	
	Artistic skills	0.269	0.152	0.696	0.491	
	Psychomotor skills	− 0.237	− 0.123	0.247	0.807	
	Outdoor activities	− 0.267	− 0.137	0.303	0.764	
	Prior experience	0.154	0.074	0.330	0.743	
Assembly	Male	0.716	0.145	0.739	0.464	0.061
	Right dominant hand	− 0.616	− 0.114	0.607	0.548	
	Laboratory + after working hours	− 0.248	− 0.049	0.241	0.811	
	Artistic skills	0.191	0.083	0.375	0.710	
	Psychomotor skills	− 0.702	− 0.283	0.554	0.583	
	Outdoor activities	− 0.198	− 0.078	0.170	0.866	
	Prior experience	0.487	0.180	0.789	0.435	
O'Connor	Male	− 1.879	− 0.059	0.297	0.768	0.038
	Right dominant hand	1.090	0.031	0.164	0.870	
	Laboratory + after working hours	1.201	0.036	0.179	0.859	
	Artistic skills	− 2.988	− 0.202	0.895	0.377	
	Psychomotor skills	3.351	0.209	0.404	0.688	
	Outdoor activities	− 0.262	− 0.016	0.034	0.973	
	Prior experience	− 2.354	− 0.135	0.583	0.563	

Note: Multivariate linear regression analysis was used.

a Significant effect at *p* < 0.05.

In relation to the direct visual test of the left hand, the right dominant hand exhibited a notable inverse effect compared with the left dominant hand ( *p* = 0.021). Moreover, additional hours spent working in the laboratory showed a substantial positive effect compared with laboratory training without extra hours ( *p* = 0.046). There was no significant impact observed on the left hand's direct visual tests in relation to gender, artistic skills, psychomotor skills, outdoor activities, and prior experience ( *p* > 0.05).

The R2 values of the regression models have been seen to be low, ranging from 0.048 to 0.1, with the greatest value of 0.36 for the left hand. This indicates that the predictors that were examined are insufficient to accurately predict the dependent variables related to direct visual perception.

**9. The effect of predictors on indirect visual test (T0 vs. T1):**

A multivariate linear regression analysis was performed to examine the impact of variables on the indirect visual test, namely, the difference between pre- and post-measurements. The predictors, such as gender, dominant hand, extra dental laboratory training, artistic skills, psychomotor skills, outdoor activities, and prior experience, did not have a significant impact on the indirect visual test for the right hand, both hands, assembly, and O'Connor Test ( *p* > 0.05; Table 10 ).

**Table 10:** Multivariate linear regression analysis to study the effect of predictors on the indirect visual test (difference between pre- and post-measurements)

	Predictors	Unstandardized coefficients	Standardized coefficients	*t* -Value	*p* -Value	*R* ^2^
Right hand	Male	0.126	0.021	0.105	0.917	0.029
	Right dominant hand	− 0.867	− 0.132	0.691	0.494	
	Laboratory + after working hours	− 0.495	− 0.080	0.389	0.699	
	Artistic skills	− 0.221	− 0.079	0.350	0.728	
	Psychomotor skills	0.435	0.144	0.278	0.783	
	Outdoor activities	− 0.504	− 0.164	0.350	0.728	
	Prior experience	0.236	0.072	0.309	0.759	
Left hand	Male	− 0.907	− 0.170	0.994	0.327	0.281
	Right dominant hand	− 2.207	− 0.501	3.798	< 0.001 a	
	Laboratory + after working hours	1.400	0.255	1.443	0.157	
	Artistic skills	0.573	0.232	1.190	0.242	
	Psychomotor skills	0.962	0.359	0.804	0.426	
	Outdoor activities	− 1.690	− 0.621	1.538	0.133	
	Prior experience	0.079	0.027	0.136	0.893	
Both hands	Male	− 0.046	− 0.010	0.050	0.960	0.102
	Right dominant hand	− 0.076	− 0.015	0.080	0.936	
	Laboratory + after working hours	− 0.284	− 0.058	0.295	0.770	
	Artistic skills	0.685	− 0.312	1.434	0.160	
	Psychomotor skills	− 0.229	− 0.096	0.193	0.848	
	Outdoor activities	− 0.563	− 0.233	0.516	0.609	
	Prior experience	− 0.005	− 0.002	0.009	0.993	
Assembly	Male	− 0.228	− 0.070	0.369	0.714	0.113
	Right dominant hand	− 0.275	− 0.078	0.425	0.673	
	Laboratory + after working hours	0.251	0.075	0.383	0.704	
	Artistic skills	− 0.084	− 0.056	0.258	0.798	
	Psychomotor skills	− 0.210	− 0.129	0.260	0.796	
	Outdoor activities	− 0.350	− 0.211	0.471	0.641	
	Prior experience	0.540	0.305	1.371	0.179	
O'Connor	Male	− 0.115	− 0.005	0.026	0.979	0.100
	Right dominant hand	2.861	0.114	0.620	0.539	
	Laboratory + after working hours	3.306	0.140	0.706	0.484	
	Artistic skills	− 1.187	− 0.111	0.511	0.613	
	Psychomotor skills	− 1.978	− 0.171	0.343	0.734	
	Outdoor activities	− 1.060	− 0.090	0.200	0.843	
	Prior experience	1.331	0.106	0.473	0.639	

Note: Multivariate linear regression analysis was used.

a Significant effect at *p* < 0.05.

Concerning the indirect visual test of the left hand, the right dominant hand exhibited a notable negative effect in comparison to the left dominant hand ( *p* < 0.001). There was no significant impact observed on the left hand's direct visual tests ( *p* > 0.05) in relation to gender, further dental laboratory training, artistic skills, psychomotor skills, outdoor activities, or prior experience. The R2 values of the regression models have been seen to be weak, ranging from a minimum of 0.029 to a maximum of 0.113, with the greatest value of 0.28 corresponding to the left hand. This suggests that the predictors or sample size being examined are inadequate for predicting the dependent variables of the direct visual test. Further investigation is needed to identify other predictors.

It is essential to underscore that the results of this study are derived from correlation and regression analyses, which discern associations but do not confirm causality. For example, although supplementary training and gender differences were correlated with enhanced manual dexterity in certain assessments, these findings must not be construed as causal relationships. To draw such conclusions with certainty, controlled experimental designs would be necessary.

## Discussion

Dental students must possess proficient manual dexterity, especially in preclinical training laboratories where they learn crucial procedures. For this study, we employed both the Purdue Pegboard Test and the O'Connor Dexterity Test to assess the improvement of manual dexterity skills and hand–eye coordination following a dental simulation preclinical training. Prior studies have shown that these assessments are capable of forecasting the academic achievement of dental students in preclinical courses. 8 9

Furthermore, research conducted by Neves et al discovered that these assessments can assess fine motor skills and hand–eye coordination, which are essential for jobs such as cavity preparations and other manual procedures in dentistry. 10

This study revealed a significant difference in the total mean scores of the Purdue and O'Connor dexterity tests when comparing the scores of all participants at T0 and T1. The results of the study done by Saeed et al were consistent with our findings. They used the Purdue Pegboard Test and the O'Connor Tweezer Dexterity Test and reported a significant improvement in students' performance on both tests after receiving preclinical training with both direct and indirect vision. 5 Unlike the study conducted by Saeed et al, our study found that students who dedicated additional time to training demonstrated significant improvement in manual dexterity abilities compared with those who did not allocate extra time. These findings suggest that practice has a substantial impact on enhancing manual dexterity skills. Similarly, a study conducted on students enrolled in phantom-head academic courses and administered assessments at three different time intervals (T0, T1, T2) demonstrated that hand dexterity may be enhanced and refined through exercise and training. This implies that by identifying students who have lower manual dexterity skills before preclinical courses, it is possible to provide specific support to help them achieve the required levels of dental performance. 2 Therefore, these results substantiate the inference that manual dexterity can be improved with regular practice and extra training.

The multivariate linear regression analysis did not reveal any statistically significant gender effect in relation to the direct and indirect visual tests conducted on the dominant hand, both hands, assembly, and the O'Connor test ( *p* > 0.05). Although previous studies have indicated possible differences in manual dexterity across genders, current research conducted by Constansia et al has found that gender does not exert a major influence on manual dexterity. This conclusion takes into consideration parameters such as finger index and thumb size when comparing men and women. 11 However, research conducted by Saeed et al found evidence of gender differences in activities involving manual dexterity. 5 12 The only significant difference in manual dexterity between genders in the present study was in the indirect visual test of the left hand, which aligns with existing literature. For instance, Psotta et al identified sex-based disparities in fine motor performance among adolescents, 12 whereas other research observed no such differences in adult cohorts. 11 This inconsistency may indicate developmental, anatomical, or sociocultural factors affecting motor learning and coordination. Further research is indicated to better understand the specific impact of gender on manual dexterity in the field of dentistry.

The correlation coefficients between visual test performance and surveyed domains in this study were found to be near zero, suggesting a weak or nonexistent relationship. Furthermore, these correlations were not statistically significant ( *p* > 0.05). These data indicate that performance on manual dexterity tests is not significantly affected by factors such as artistic skills, psychomotor skills, outdoor activities, or past experience as measured by the survey. Johnson et al discovered a positive correlation between higher preclinical practical scores and increased engagement in psychomotor, artistic, and outdoor physical activities. Notwithstanding these findings, they proposed that engagement in these activities cannot serve as a reliable indicator for the performance of dental students. This is because they found that not all individuals with a high level of participation excelled in practical exams, and not all individuals who excelled in practical exams had high levels of participation in the activity factors. 6

The study examined the influence of variations in dominant hand preference among persons who are left-handed or right-handed on the outcomes of dexterity tests, namely, the Purdue Pegboard Test and O'Connor Tweezer Test. The results indicated that, on the whole, variations in dominant hand did not have a significant impact on the test outcomes. However, there was a substantial deviation from the norm in the Purdue DVT test of the left hand. Left-handed participants displayed considerably higher scores compared with their right-handed counterparts ( *p* = 0.004). This implies that persons who are left-handed possess proficient manual dexterity in both their dominant and nondominant hands. Similarly, Judge and Stirling observed a significant enhancement in the performance of left-handed individuals while participating in tasks that necessitate coordination between their left and right hands, as demonstrated by the Purdue Pegboard Test. 13 A supplementary study revealed a correlation between handedness and performance on the Purdue Pegboard Test, indicating that left-handed individuals occasionally exhibit superior performance compared with right-handed individuals in specific activities. 14

However, there is a lack of research examining the impact of left- and right-dominant hands on manual dexterity outcomes in dentistry, particularly the effects of being left-handed on manual dexterity and clinical practice. A recent study has uncovered that dentistry students who are left-handed may face challenges when adapting to dental tools that are primarily developed for right-handed individuals, as well as when performing certain dental procedures. 15 Therefore, dental schools should take into consideration the distinct needs of left-handed students to foster inclusion and improve their performance in clinical practice.

The superior performance of left-handed individuals in the left-hand direct visual test corresponds with the observations of Judge and Stirling, who identified benefits for left-handers in tasks necessitating bimanual coordination. 13 Nonetheless, these findings are still limited and need more research in the context of dental education, especially since left-handed students may have trouble using mostly right-handed tools and workstations.

## Study Limitations

A small sample size from one institution may introduce selection bias and limit generalizability. Based on self-reported questionnaires, data gathering may have been biased and inaccurate. Due to the study's short follow-up, socioeconomic status and cognitive abilities were not sufficiently accounted for, which may have affected the results' validity.

Another notable limitation is the low *R* ^2^ values yielded by the regression models, indicating that the included predictors had limited power in explaining the variance in manual dexterity scores. This suggests the presence of other influential factors not captured by the survey or study design. Furthermore, the correlation-based methodology inherently limits the ability to draw causal conclusions from the observed associations. Lastly, the significant findings related to gender and handedness should be cautiously interpreted in light of limited sample diversity and the potential influence of confounding variables not assessed in this study.

## Conclusion

This study provides evidence of the positive impacts of preclinical training on the manual dexterity skills of dental students. The results show significant increases in both the Purdue Pegboard Test and the O'Connor Tweezer Dexterity Test after just one semester. The existence of gender differences in performance indicates the need for additional research and exploration. To improve the applicability of future research findings and investigate the long-term consequences, it is recommended to utilize larger and more diverse participant samples, as well as longitudinal study methods. It is advisable to use comprehensive tests, in addition to self-reported questionnaires, to obtain a complete picture of manual dexterity in dentistry education. These observations can enhance the development of the curriculum and enhance the readiness and confidence of dentists.
